# Diagnosis characteristics and therapeutical options of infectious complications associated with peritoneal dialysis

**Published:** 2014

**Authors:** O Mihalache, H Doran, E Catrina, F Bobircă, P Mustatea, D Georgescu, T Pătrașcu

**Affiliations:** * ”Carol Davila” University of Medicine and Pharmacy, Bucharest, Romania; ** Surgical Clinic I, “Juvara” Clinical Hospital; “Dr. I. Cantacuzino” Hospital, Bucharest, Romania

**Keywords:** peritoneal dialysis, peritonitis, tunnel infections, ultrafiltration failure

## Abstract

**Introduction**. The infectious syndrome associated with peritoneal dialysis is the most important complication of this substitution method of the renal function, also being the main cause of method failure. Refractory peritonitis can cause real problems in the differential diagnosis with secondary peritonitis, which can delay the surgical intervention and endanger the patient’s life.

**Materials and methods**. The patients with an end stage renal disease under peritoneal dialysis, who were admitted to “I. Juvara” Surgical Clinic of “Dr. I. Cantacuzino” Clinical Hospital, between 2007 and 2011, were retrospectively analyzed for catheter removal/ replacement due to infectious complications or ultrafiltration failure.

**Results**. 55 patients were identified: 33 with infectious complications (exit-site, tunnel infections 4 and peritonitis 29) and 22 with loss of peritoneum ultrafiltration capacity. The patients with ultrafiltration failure had a longer duration of PD and a smaller number of peritonitic episodes (0.28 episodes/ year at risk in the ultrafiltration failure group vs. 0.98, in the group of infectious complications). The removal of the catheter was the only surgical procedure performed for the patients with ultrafiltration failure, while the patients with peritonitis needed additional gestures like an exploratory laparotomy with peritoneal lavage and drainage and adhesiolysis in the majority of cases. In the group with infectious complications, 4 patients died: 2 by multisystem organ failure due to prolonged sepsis, one developed an upper gastrointestinal bleeding followed by respiratory insufficiency and one had in cataclysmic gastrointestinal bleeding which rapidly led to death.

**Conclusions**. The immediate operative approach for an infectious peritoneal syndrome under peritoneal dialysis is seldom necessary. The surgical observation is absolutely mandatory in every case. The absence of a response to the proper medical treatment is an indication of peritoneal cavity exploration including laparoscopy/ laparotomy. Any delay in the diagnosis and definitive treatment gives an extremely high mortality rate.

## Introduction

Peritoneal dialysis (PD) is associated with a high risk of infection of the peritoneum, subcutaneous tunnel and exit-site. Infectious complications generate increased morbidity and mortality rates, being the main cause of failure in PD [**1**]. Peritonitis remains the leading complication having around 18% of the infection-related mortality in PD patients. Although less than 4% of the peritonitic episodes result in death, peritonitis is a “contributing-factor” in 16% of the deaths on PD [**2**]. The majority of catheter related problems are of an infectious nature- mainly represented by peritonitis (61%), exit-site and tunnel infections (23%), catheter obstruction, dislocation and leakage making up the rest [**3**]. It is estimated that 12% of the cases of exit-site and tunnel infections result in PD peritonitis [**4**]. Usually, it has an excellent prognosis with a resolution within days, but can be associated with a severe pain leading to hospitalization, catheter loss and a risk of death; it can also occasionally lead to much dreaded encapsulating peritoneal sclerosis. Peritonitis treatment should aim for a rapid resolution of inflammation and preservation of peritoneal membrane function [**8**]. The action to decrease the risk of infections associated with PD should start in the pre-catheter insertion phase. In order to obtain a reduction of the complications, achieve prolonged catheter duration and a better quality of life for PD patients, the surgical technique requires a strict adherence to a standardized procedure and dedicated team [**5**].

## Material and Methods

The patients with an end stage renal disease under peritoneal dialysis, who were admitted in “I. Juvara” Surgical Clinic of “Dr. I. Cantacuzino” Clinical Hospital, between 2007 and 2011 for catheter removal/ replacement due to infectious complications or ultrafiltration failure, were retrospectively analyzed. Peritonitis diagnosis was based on the presence of at least 2 of the following: cloudy effluent, abdominal pain and positive culture from dialysate. Exit-site and tunnel infections were diagnosed on the presence of inflammation signs and purulent drainage. Patients were compared to see if there is any correlation between age, sex, level of education, duration of PD, number of peritonitic episodes and comorbidities. In the group with infectious complications, we also studied the type of peritonitis, microbiological culture, surgical procedure that was performed.

## Results

55 patients were identified: 33 with infectious complications (exit-site, tunnel infections 4 and peritonitis 29) and 22 with loss of peritoneum ultrafiltration capacity. The diagnosis of infectious complications (IC) and ultrafiltration failure (UF) was done in the dialysis center by using the criteria described in methods. The mean age, sex distribution, peritoneal dialysis duration, mean hospital stay, provenience area, level of education and comorbidities are presented in **Table 1**

**Table 1:** Demographic data of the patients with infections complication and ultrafiltration failure. *Chi square test

		Infectious complications of PD	Ultrafiltration failure	P value*
Number of patients		33	22	
Sex	Male n, (%)	22 (66.6)	14 (63.6)	1
	Female n, (%)	11 (33.3)	8 (36.4)	
Mean age (years)		60.6 ±12.5	59.22±12.5	
Mean time on PD (months)		29.48	39.40	
Mean hospital stay (days)		5,3	1,9	
Area of provenience	Urban	19	20	0.007
	Rural	14	2	
Level of education	grade school	16	6	0.27
	High school	12	12	
	College	5	4	
Comorbidities	Diabetes	21	17	0.59
	Cardiovascular	17	8	
	Neurological	4	3	

The mean age of the patients with IC was similar to that of the patients with UF. Sex ratio was 2 male to 1 female in the group with infectious complications and respectively 1.75 to 1 in the group with ultrafiltration failure. The patients with UF had a longer duration of PD and a smaller number of peritonitic episodes (0.28 episodes/ year at risk in the UF group vs. 0.98 in the group of IC). The removal of the catheter was the only surgical procedure performed for the patients with UF, while the patients with peritonitis needed additional gestures like an exploratory laparotomy with peritoneal lavage and drainage in 17 out of 33 cases. Also adhesiolysis was performed in 9 cases. In the 4 cases of exit-site and tunnel infections, surgical drainage was performed in 3 cases, and the replacement of the catheter in one case. The indications for catheter removal in cases with peritonitis were the refractory, relapsing or recurrent peritonitis or identification of fungi in the dialysate. Types and etiology of peritonitis are summarized in **Table 2**. There were 3 secondary peritonitis: 2 acute cholecystitis and a pelvic abscess due to sigmoidian diverticulitis with ischemic enteritis. The first two cases were solved by cholecystectomy along with the catheter removal and the third, by drainage of the abscess. 

**Table 2:** Type and etiology of peritonitis

Etiology	Type of peritonitis	Refractory	Relapsing	Recurrent	Secondary
Bacterial		4	1		
Fungal		8	1		1
Sterile		2	3		
Without microbiological culture		3		4	2
Total		17	5	4	3

The mean hospital stay was higher for the patients with IC. Postoperatively, there were no complications in the group with UF. In the group of patients with infectious complications, one patient developed a postoperative bowel obstruction, which needed a reintervention with a favorable evolution afterwards; one patient had a mechanical malfunction of the catheter, which was solved by laparoscopic repositioning of the catheter. 4 patients died: 2 by multisystem organ failure due to prolonged sepsis, one developed an upper gastrointestinal bleeding followed by respiratory insufficiency an one had a cataclysmic gastrointestinal bleeding which rapidly led to death. 

## Discussion

For the PD program to be successful, close attention must be paid to the prevention of the infectious complications. Those are defined as exit-site infections, tunnel infections and peritonitis.

Exit-site infections are defined by the presence of purulent drainage, with or without erythema of the skin at the catheter-epidermal interface. Pericatheter erythema of the skin without purulent drainage is sometimes an early indication of infection [**2**,**6**,**9**]. Tunnel infections may present erythema, edema or tenderness over the subcutaneous pathway but are often clinically occult. Usually, tunnel infections occur in the presence of an exit-site infection, but rarely alone. The diagnostic of these infections is mainly based on clinical signs. The ultrasound examination of the catheter tract can be useful for the diagnosis of the tunnel infections. An area of hyperechogenicity of more than 2 mm in width along any portion of the catheter tract is considered a positive finding. The most common pathogens are Staphylococcus Aureus and Pseudomonas aeruginosa [**3**,**7**,**9**]. Such infections must be treated aggressively, oral antibiotic therapy being generally recommended. Catheter removal is required if an exit-site of the tunnel infection is presented in conjunction with peritonitis. The simultaneous removal and reinsertion of the dialysis catheter (with a new exit-site) is feasible in eradicating refractory infections due to P. aeruginosa. In selected cases, cuff shaving may be considered an alternative to catheter replacement for tunnel infections [**1**,**2**].

Peritonitis in PD patients has important diagnostic, progression and treatment features, which separate them from the “classical” surgical peritonitis [**1**]. The main clinical sign is the cloudy aspect of the effluent. This will usually represent infectious peritonitis but other causes like chemical peritonitis, eosinophilia of the effluent or malignancy may present with this aspect. Abdominal pain is the second clinical element, which should include peritonitis in the differential diagnosis of the patients on PD. The degree of pain can vary from severe to mild or even no pain and is somewhat an organism specific (generally less with coagulase-negative Staphylococcus and grater with Streptococcus, gram-negative and S. aureus). The confirmation of the diagnostic is made by obtaining a cell count form the dialysate and a positive microbiological culture. For the positive diagnostic, 2 of the following 3 are necessary: cloudy effluent, cell count with white blood cells more than 100/µl and at least 50% polymorphonuclear neutorphilic cells and positive culture from the dialysate [**2**,**4**]. The treatment must be started empirically prior to the knowledge of the causative organism and consist in broad-spectrum antibiotics administrated intraperitoneally or intravenously. Intraperitoneal administration is superior to IV administration due to the possibility of achieving a higher concentration at the site and a lower toxicity [**2**]. The therapeutic recommendations under the auspices of International Society for Peritoneal Dialysis (ISPD) were first published in 1983 and revised five times, the last revision having taken place in 2005. With the appropriate treatment, resolution should be obtained in a few days. Failure of the effluent to clear after 5 days of appropriate antibiotics defines a refractory peritonitis, which should be managed by the removal of the catheter to protect the peritoneal membrane. The other indications for catheter removal are the following: refractory peritonitis, relapsing peritonitis, fungal peritonitis (**Table 3** terminology for peritonitis). 

**Table 3:** Terminology for Peritonitis

Recurrent	An episode that occurs within 4 weeks from the completion of therapy of a prior episode but with a different organism
Relapsing .	An episode that occurs within 4 weeks from completion of therapy of a prior episode with the same organism or 1 sterile episode
Repeat	An episode that occurs more than 4 weeks after the completion of therapy of a prior episode with the same organism
Refractory	Failure of the effluent to clear after 5 days of appropriate antibiotics
Catheter-related peritonitis	Peritonitis in conjunction with an exit-site or tunnel infection with the same organism or 1 sterile site

For the surgeon, the real challenge is the differential diagnosis of refractory and secondary peritonitis. The dilemma must be solved quickly with a careful and complete imagistic exploration, searching to eliminate intraperitoneal loculated collections, digestive and genital associated pathology. An incorrect diagnostic and a delayed correct treatment may have severe consequences for the patient outcome exploratory laparotomy, or laparoscopy is indicated to all patients with persistence of peritonitis signs after or a failure to improve after 5 days of correct antibiotic therapy [**1**].

## Conclusions

The immediate operative approach for an infectious peritoneal syndrome under peritoneal dialysis is seldom necessary, in the majority of peritonitis related to peritoneal dialysis, the source of contamination is external – “non-surgical”. The surgical observation is absolutely mandatory in every case. The absence of a response to the proper medical treatment is an indication of peritoneal cavity exploration including laparoscopy/ laparotomy. Any delay in the diagnosis and definitive treatment gives an extremely high mortality rate.
